# Count trends for migratory Bald Eagles reveal differences between two populations at a spring site along the Lake Ontario shoreline

**DOI:** 10.7717/peerj.1986

**Published:** 2016-05-17

**Authors:** Kyle R. Wright

**Affiliations:** Onondaga Audubon Society, Mexico, NY, United States

**Keywords:** Bald eagle, Haliaeetus leucocephalus, Migration counts, Population trends, Migration ecology, Population ecology

## Abstract

The recovery of Bald Eagles (*Haliaeetus leucophalus*), after DDT and other organochlorine insecticides were banned in the United States, can be regarded as one of the most iconic success stories resulting from the Endangered Species Act. Interest remains high in the recovery and growth of the Bald Eagle population. Common to evaluating growth and recovery rates are counts at nesting sites and analyses of individuals fledged per season. But this is merely one snapshot that ignores survival rates as eagles grow to maturity. By analyzing indices from migration counts, we get a different snapshot better reflecting the survival of young birds. Different populations of Bald Eagles breed at different sites at different times of the year. Typical migration count analyses do not separate the populations. A separation of two distinct populations can be achieved at spring count sites by taking advantage of the tendency for northern summer breeding birds to migrate north in spring earlier than southern winter breeding birds who disperse north later in spring. In this paper I analyze migratory indices at a spring site along Lake Ontario. The analysis shows that eagles considered to be primarily of the northern summer breeding population showed an estimated growth rate of 5.3 ± 0.85% (SE) per year with 49% of eagles tallied in adult plumage, whereas the migrants considered to be primarily of the southern breeding population had an estimated growth rate of 14.0 ± 1.79% with only 22% in adult plumage. Together these results argue that the populations of southern breeding Bald Eagles are growing at a substantially higher rate than northern breeding eagles. These findings suggest that aggregate population indices for a species at migration counting sites can sometimes obscure important differences among separate populations at any given site and that separating counts by time period can be a useful way to check for differences among sub-populations.

## Introduction

Data from raptor migration counts can provide scientists and managers insight into a population’s well-being and dynamics (Kerlinger, 1989; Goodrich & Smith, 2008). Many papers analyze the variable numbers of the Bald Eagle (*Haliaeetus leucophalus*) based on migration counts in the second half of the 20th century (e.g., Bednarz et al., 1990; Farmer et al., 2008; Farmer & Smith, 2010). Among the most comprehensive, the landmark 2008 State of North America’s Birds of Prey (Bildstein et al., 2008) contains analyses of migration counts based largely on fall sites, and Farmer and Smith’s 2010 follow up uses similar statistics to analyze spring migration data and indices. These studies, like many others, while quite robust in some respects, fail to do much analysis on the two distinctive populations of Bald Eagles encountered at many migration count sites in the Mid-Atlantic and Northeastern regions of the United States. Failure to separate the analysis of the two populations can potentially mask evidence regarding the well-being of one or both populations. This paper contains an investigation into the consequences of separating the analysis of the two populations at one spring migration site.

Historically, two subspecies of Bald Eagle were recognized by some authors (Stalmaster, 1987; Buehler, 2000). Differentiation between the subspecies was based loosely on breeding range and a few morphometric measurements (Stalmaster, 1987; Nye, 1998; Buehler, 2000; Wheeler, 2003). The latter, however, were clinal in nature and were distorted by the hacking and reintroduction programs of the 1970s and 1980s (Stalmaster, 1987; Nye, 1998; Buehler, 2000; Wheeler, 2003). The subspecies were never defined based on breeding timing or the nature and timing of movements away from the breeding grounds. Today, most authors do not regard the species as polytypic (i.e., Nye, 1998; Wheeler, 2003). Nevertheless, Bald Eagles occurring east of the Mississippi River and north into Eastern Canada can be classified into two separate populations based on timing and location of breeding activities and timing and nature of nonbreeding movements. Birds breeding in the northeastern parts of the species’ range disperse south in the months of October–January and return north in the months of February–April (Wheeler, 2003). In contrast, much like an austral migrant but in the northern hemisphere, Bald Eagles breeding in the southeastern United States from the Texas Coast to the Carolinas disperse northwards from February to June after a winter breeding season (Broley, 1947; Wheeler, 2003).

Considerable discussion takes place on hawkwatch lookouts throughout the Northeastern United States and Mid-Atlantic States about migration timing and whether observed eagles belong to northern or southern populations. These speculations have even made it into a number of hawkwatch’s end of season reports. To date, however, no peer-reviewed publication has attempted to conduct separate analyses for the two populations based on migration counts. Separating data for birds belonging to northern and southern populations may shed light on differences in the ecology and growth of the two populations. In contrast, limiting all migratory analysis to combined data could mask substantial differences in growth trends and overall well-being of the two populations. Specifically, the smaller of the two populations could show evidence of slow growth or even decline in well-being that would not be recognized by focusing exclusively on the combined data. Even though Bald Eagles were recently declassified as an endangered species, interest remains high in maintaining healthy populations. Presumably, that interest would apply to both the northern and the southern population. This paper is a first step in addressing that knowledge gap. Using data from spring counts at Derby Hill Bird Observatory, I analyze separate growth rates for the two populations. In addition, I consider the ratio of adult birds to the total number of birds for each population, with lower percentages of adult birds typically regarded as evidence of a population in steeper growth.

## Materials and Methods

### Count site and data collection

Derby Hill Bird Observatory (DHBO) in Mexico, New York, United States, is located on the southeast corner of Lake Ontario (N43°31′39″, W76°14′22″). The site is at the extreme eastern end of the diversion line created by the south shores of Lakes Erie and Ontario and therefore acts as a significant funneling and concentrating point for migrating raptors in the spring season (Haugh & Cade, 1966; Mueller & Berger, 1967). For this reason, standardized surveys of migrating raptors have occurred annually at the DHBO site since 1979, with the count season typically starting in late February or early March and continuing through the end of May. The days and hours of coverage are determined by the lead counter, and vary according to weather conditions. Counts are conducted from one of two lookouts approximately one and a half kilometers apart. Each east/northeast bound passing raptor is identified to species and tallied, with additional data including classifications of age, sex, and/or color morph on select birds collected at the individual counter’s discretion.

For this study, the author separated the observations of Bald Eagles into northern and southern populations based on the timing of the passage. As noted in the introduction, Bald Eagles that breed in the north will disperse to the south during late fall and early winter and return north in early spring, typically passing over DHBO from February to April. Eagles that breed in the winter months in the southeast will disperse northward after their breeding season and will typically pass over DBHO in May. One cannot assume that there is an absolute split between the passage of the northern and southern populations. Actual migration patterns will vary based on a number of factors including breeding success, weather, and prey availability (Buehler, 2000). For the purpose of this study, the author classified Bald Eagles flying over DHBO before April 1 as northern breeding birds and birds flying over after May 10 as southern breeding birds. For the remainder of this and the results sections, all references to northern breeding eagles are in fact references to eagles passing over DHBO in the earlier period. Similarly, references to southern breeding eagles are references to eagles passing over DHBO in the later period. A few eagles that breed in the south could be among the birds labeled as northern breeding and vice versa. However, the conservative classification (while ignoring Bald Eagles observed between April 1 and May 10) increases the likelihood that the classified birds will belong to the correct population.

In addition to classifying birds by time of passage, this study contains analyses of the difference in proportions of the adults for the two different populations. Reliable Bald Eagle age classification data were collected in 1994–1997, 2009, 2012–2013, and 2015. Consequently, the proportion of adult analysis is limited to those time periods.

### Statistical analysis

All statistical analysis was performed with *R version 3.2.0*. With the first analysis, the author focused on determining whether or not there is a significant difference in the growth rate for the population identified as northern versus the population identified as southern. Population growth rates were determined utilizing data covering the 25-year span from 1991 to 2015. Using the classifications of northern and southern, as described above, the author calculated a bird per hour number by dividing the total birds counted in the identified time period by the total hours of observation for the same period. Using the birds per hour data, the author created an exponential regression model for each population by applying a natural log transformation to each annual value (birds per hour) and fitting a linear regression model to the transformed data. Once growth rates were determined, the author applied a *t*-test to determine whether or not there was a significant difference between the two growth rates. To consider the extent to which a single analysis of a whole season’s worth of data might mask important information about a smaller individual population, the author also created an exponential regression model for the entire Bald Eagle per hour count over the same time period. Again *t*-tests were used to evaluate the difference in the resulting growth rates.

In the second analysis, the author focused on the proportion of adults. For the eight years in which reliable age classifications exist, the author calculated the proportion of the population comprised of adults for the northern and southern populations. In each case, this proportion was obtained by dividing the total number of identified adults over all eight years by the total number of aged birds for each temporal window. In order to determine if there is a significant difference in these two proportions, the author applied a two-proportion *t*-test for the difference in proportions with a continuity correction.

In order to determine whether or not the proportion comprised of adults has changed over time, the author calculated, for both populations, the proportion comprised of adults for the four years up to 1997, and the proportion comprised of results for the years after 2008. Again a two-proportion *t*-test for the difference in proportions with a continuity correction was applied to determine if a significant difference can be observed between the two time periods for each population.

## Results

The first set of results provides separate growth rates for the northern and southern populations. The exponential model for northern eagles per hour for the 25-year window from 1991 to 2015 is

\begin{eqnarray*}y=0.106{e}^{0.053x} \end{eqnarray*}

where *y* = the number of birds observed per hour and *x* = the period (1,…,25) and 0.053 represents the annual growth rate over the 25-year period.

For the southern population, the exponential model for eagles per hour for the 25-year window from 1991 to 2015 is

\begin{eqnarray*}y=0.094{e}^{0.140x} \end{eqnarray*}

where *y* = the number of birds observed per hour and *x* = the period (1,…,25) and 0.140 represents the annual growth rate over the 25-year period.

**Figure 1:**
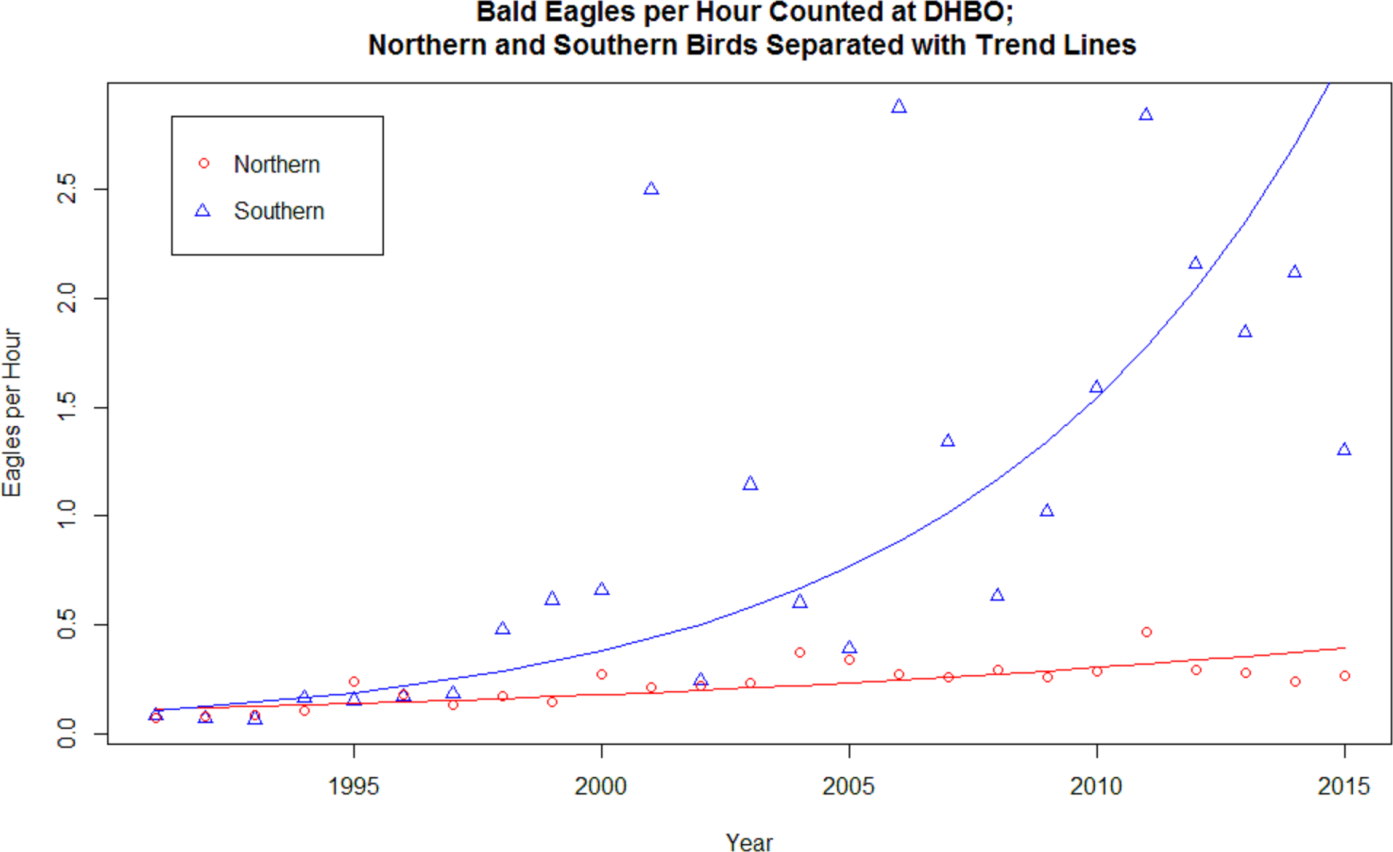
Bald Eagles per hour counted at DHBO; northern and southern birds separated with trend lines. Bald Eagles per hour of observation recorded at Derby Hill Bird Observatory, 1991–2015. “Northern” birds, those counted before 1 April, represented by red circles. “Southern” birds, those counted after 10 May, represented by blue triangles. Curves for exponential models of best fit for each population also included: the northern model in red and the southern model in blue.

Figure 1 contains a graph of the data values for both populations and the resulting exponential model in each case. Table 1 contains the results from the *t*-test for the difference in the growth rates for the two populations, as well as the Adjusted R Square for each model. The results indicate that there is clear evidence of a significant difference in these growth rates. In fact, the 95% confidence interval for the difference in growth rates has a lower endpoint in excess of 0.04, indicating that there is evidence that the difference in growth rates exceeds this amount.

**Table 1:** Northern vs. Southern Bald Eagle growth rates. Test for difference in growth rates in assumed northern and southern breeding populations based on DHBO data.

Assumed population	Growth rate	Adjusted R square	Difference in growth rates	*P*-Value significance test for difference	95% confidence interval for difference
Northern	0.053	0.62	0.088	0.000063	(0.048 , 0.128)
Southern	0.140	0.71			

For the entire seasons’ data, the exponential model for eagles per hour for the 25-year window from 1991 to 2015 is

\begin{eqnarray*}y=0.0735{e}^{0.118x} \end{eqnarray*}

where *y* = the number of birds observed per hour and *x* = the period (1,…,25) and 0.118 represents the annual growth rate over the 25-year period.

Tables 2 and 3 contain the results from the *t*-test for the differences in growth rates for the temporal windows of presumed northern and southern populations when compared to data for the full seasons. The results in Table 2 indicate that there is not sufficient evidence to conclude that there is a difference between the growth rate for the southern breeding population and the growth rate for the entire seasons’ worth of birds. In contrast, the results in Table 3 indicate that there is evidence of a significant difference between the growth rate for the northern breeding population and the entire seasons’ worth of birds.

**Table 2:** Whole season vs. Southern only bald eagle growth rates. Test for difference in growth rates based on DHBO data between temporal windows for assumed southern breeding populations and full seasons aggregate data.

Assumed population	Growth rate	Adjusted R square	Difference in growth rates	*P*-value significance test for difference	95% confidence interval for difference
Southern	0.140	0.71	0.022	0.271000	(−0.062 , 0.018)
All Birds	0.118	0.89			

**Table 3:** Whole season vs. northern only bald eagle growth rates. Test for difference in growth rates based on DHBO data between temporal windows for assumed northern breeding populations and full seasons aggregate data.

Assumed population	Growth rate	Adjusted R square	Difference in growth rates	*P*-value significance test for difference	95% confidence interval for difference
Northern	0.053	0.62	0.065	0.000002	(0.041 , 0.089)
All birds	0.118	0.89			

The second set of results provides the proportion of adults for each population. For the northern population, the proportion comprised of adults over the full eight years is 0.489. For the southern population, the proportion comprised of adults over the full eight years is 0.223. Table 4 contains the results for the test for the difference in the proportions. The results clearly indicate that there is a significant difference between the proportion of adults for the two populations. Furthermore, the lower end of the 95% confidence interval for the difference in proportions provides evidence that the difference exceeds 0.199. As illustrated in Tables 5 and 6, the analysis of the proportions comprised of adults before 1998 and after 2008 failed to provide evidence of a difference in proportions for either population.

**Table 4:** Northern vs. southern bald eagle proportion adults. Test for difference in adult proportions for northern population and southern population.

Assumed population	Number aged	Number adults	Adult proportion	Difference in proportions	*P*-value significance test for difference	95% confidence interval for difference
Northern	266	131	0.49	0.270	1.47E–15	(0.199, 0.341)
Southern	642	143	0.22			

**Table 5:** Pre 1998 vs. post 2008 northern proportion adults. Test for difference in adult proportions for northern population pre 1998 vs. post 2008.

Time period	Number aged	Number adults	Adult proportion	Difference in proportions	*P*-value significance test for difference	95% confidence interval for difference
Pre 1998	98	54	0.55	0.09	0.183	(−0.039, 0.225)
Post 2008	168	77	0.46			

**Table 6:** Pre 1998 vs. post 2008 southern proportion adults. Test for difference in adult proportions for southern population pre 1998 vs. post 2008.

Time period	Number aged	Number adults	Adult proportion	Difference in Proportions	*P*-value significance test for difference	95% confidence interval for difference
Pre 1998	46	8	0.17	0.05	0.521	(−0.179, 0.074)
Post 2008	596	135	0.23			

## Discussion

The results of this analysis show that, over the twenty-five year period from 1991 to 2015, the annual growth rate for Bald Eagles passing over DHBO before the end of March is 5.3% while the growth rate for Bald Eagles passing over DHBO after May 10 is 14% and that the difference between these two rates is statistically significant (Table 1). The author conservatively selected the time periods used in analysis so that the February–March time period would likely include only Bald Eagles that were returning to their northern breeding grounds. Southern winter breeding Bald Eagles would be highly unlikely to have made it this far north by the end of March (Broley, 1947; Buehler, 2000; Wheeler, 2003). Similarly, Bald Eagles on their way to north to breed during summer months are likely passing over DHBO in the months of February, March and April, and are unlikely to be making that passage at late as May 11 (Buehler, 2000; Wheeler, 2003). Consequently the time period after May 10 would likely include on southern breeding Bald Eagles. Hence there is reasonable evidence, at least for Bald Eagles passing over DHBO, that the northern breeding population of Bald Eagles is growing at a substantially slower rate that the southern breeding populations.

Inherent in any analysis of a population index inferred from a migration count is the assumption that the percent of the population sampled is similar from year to year (Kerlinger, 1989). The February and March time period does not include all of the northern breeding birds passing over DHBO; some individuals may still be trickling through in early April. There is the possibility that changes in climate conditions have caused a change in the time of passage and hence is an alternate explanation for low growth rates. However, climatic changes would most likely result in summer breeding Bald Eagles making their way to the northern breeding sites earlier and hence would actually increase the proportion of northern breeding birds passing over DHBO during February and March as opposed to April. That scenario would lead to a growth rate that overestimates the actual growth rate of the northern breeding population.

A more likely consequence of any climatic changes could be a shift in geographic migratory patterns resulting in fewer northern breeding birds passing over DHBO on the way to their breeding sites. Such a change may result in a different sample of a different population of Bald Eagles passing over DHBO in a similar fashion to the theory termed “migratory short stopping” proposed for Sharp-shinned Hawks (*Accipiter striatus*) by Viverette et al. (1996). The only way this possibility of shifts in migration routes and therefore population sampling consistency can be investigated is to look at the data collected at other migration count sites in conjunction with sampling on the breeding and non-breeding grounds. With the standard analysis of growth rates for Bald Eagles at Mid-Atlantic and Northeastern migration sites not differentiating between the two populations, the question of shifts in migratory patterns on geographic scales cannot be addressed. Furthermore, the results of this study show that the growth rate for all Bald Eagles passing over DHBO is not significantly different from that of the southern breeding population (Table 2) but that is it significantly and substantially different from that of the northern breeding population (Table 3). Continuing to evaluate the well-being of Bald Eagles based on the total populations can potentially mask ecological challenges faced by the northern breeding population.

Eagles passing over DHBO between April 1 and May 10 were not included in this study. Conservative temporal windows were deemed a priority in this study. However, the ability to include more of these “missed” birds could enhance the quality of any analysis. As noted in the methods section, the passage time for each subpopulation can vary according to number of factors including breeding success, weather, and prey availability (Buehler, 2000). An attempt could be made to annually adjust the time periods based on known weather factors. Methods for separating populations such as cluster analysis could allow for defining time periods that would vary year to year and potentially include more eagles. Additionally, as understandings of molt, plumage progression, and feather wear and fade advance, in conjunction with heightened observer skill and optical advancements, separating winter from summer hatched eagles in the field based on plumage and molt characteristics may become more and more feasible.

In addition to establishing a significant difference in growth rates for birds passing over DHBO in the two time periods, the results of the study show that the adult proportion of Bald Eagles observed in the time period prior to the end of March was 0.489 while the adult proportion for birds passing over after May 10 was 0.223 (see Table 4). These differences in percent adult birds between the northern and southern Bald Eagles are consistent with general understandings of the migratory patterns of the two populations. Broley (1947) notes that adult Bald Eagles are present in Florida in the months of May and September but are virtually absent in the months of July and August. Buehler (2000) goes even further to state that “Southern adults (breeding south of 40°N) usually do not migrate but remain year-round in the vicinity of the nest site.” Both of these positions are consistent with a smaller proportion of adult birds from the southern breeding population passing over DHBO. In addition, the proportion of adults is impacted by the high first year mortality in many migratory avian species (Owen & Black, 1989; Menu, Gauthier & Reed, 2005). Cohorts of the previous summer’s hatched Bald Eagles passing DHBO in the spring are already on their second trans-latitudinal migration and have already been thinned by a first winter experience. This thinning of the cohort has not yet happened to winter hatched birds headed north past DHBO in the spring. This theory is supported with DHBO numbers. In 2009 and 2012 more specific age classes were assigned to many of the non-adult Bald Eagles. In February and March 2009 and 2012, 40% of the aged non-adult birds were less than one year out of the nest. For the same two years post-May 10, 91% of the aged non-adult birds were less than one year out of the nest. The impact of the first year mortality rates is illustrated by these differences in the percent of less than one-year-old non-adults. This is consistent with larger portions of adult birds recorded in the temporal window for the northern breeding population. Hence, both the migration patterns for southern breeding adults and the impact of first winter mortality rates are consistent with the statistical results of this study which show a significant and substantial lower proportion of adults in the birds passing over DHBO after May 10. This makes it even more reasonable to assume that these later passing Bald Eagles represent a southern breeding population and the earlier passing Bald Eagles representing a northern breeding population.

This paper provides evidence that two differently behaving groups of Bald Eagles pass over DHBO in two distinct temporal windows and that the annual growth rate for one is substantially lower than that of the other. In conjunction with current knowledge of the species’ breeding biology and migratory habits, it is not an unreasonable conclusion that these two groupings are representative of two separate populations of Bald Eagles whose conservation status and management should perhaps be treated separately. Migration monitoring gives important snapshots into a population’s wellbeing not obtained from breeding bird surveys or surveys of birds dispersed to nonbreeding grounds. More research and analysis of the two populations of Bald Eagles at additional migration count sites throughout the Mid-Atlantic and Northeastern United States in spring and fall could lead to better conservation practices for the species in addition to more insights into the impacts of a changing environment.

## Additional Information and Declarations

### Data Availability

The following information was supplied regarding data availability: The data presented in this paper are the property of Onondaga Audubon Society. Requests for data made be made to the Onondaga Audubon Society, 36 GrandView Ave, Mexico, NY 13114, United States or onauduboncom@gmail.com.
